# Immune‐Related Polyradiculoneuropathy Associated With Immune Checkpoint Inhibitors: A Comprehensive Case Series

**DOI:** 10.1002/brb3.71440

**Published:** 2026-04-28

**Authors:** A. Llauradó, E. Lainez, H. Ariño, M. Gratacòs‐Viñola, J. L. Seoane, M. Roca‐Herrera, J. L. Restrepo‐Vera, D. Sanchez‐Tejerina, N. Raguer, R. Juntas‐Morales

**Affiliations:** ^1^ Neuromuscular Disorders Unit, Department of Neurology Vall D'hebron University Hospital Barcelona Spain; ^2^ Department of Clinical Neurophysiology Vall D'hebron University Hospital Barcelona Spain; ^3^ Neurology Department and Centre d'Esclerosi Múltiple de Catalunya (Cemcat) Vall D'hebron University Hospital Barcelona Spain; ^4^ Department of Oncology Vall D'hebron University Hospital Barcelona Spain

**Keywords:** clinical outcome, Guillain–Barré‐like syndrome, immune checkpoint inhibitors, immune‐mediated neurological adverse events, immune‐related polyradiculoneuropathy

## Abstract

**Objective:**

Immune‐related polyradiculoneuropathy (irPRN) is a rare but potentially severe neurological adverse event secondary to immune checkpoint inhibitors (ICIs), closely resembling Guillain–Barré syndrome. This study aims to characterize the clinical presentation, neurophysiological findings, therapeutic strategies, and clinical outcomes of patients diagnosed with irPRN.

**Methods:**

We conducted a retrospective single‐center study including patients diagnosed with irPRN following ICI therapy between May 2018 and July 2024. Clinical, electrophysiological, and cerebrospinal fluid (CSF) data were analyzed.

**Results:**

Nine patients (mean age 67.9 years; 66.7% male) developed immune‐related polyradiculoneuropathy (irPRN), with a median onset of 6 weeks following initiation of immune checkpoint inhibitors (ICIs). Most cases (66.7%) occurred in patients receiving anti‐PD‐1 monotherapy. The clinical presentation was characterized by symmetric limb weakness (100%) and sensory disturbances (88.9%). Electrodiagnostic studies demonstrated a demyelinating polyradiculoneuropathy in 8 of 9 patients; only one exhibited an axonal pattern. CSF analysis revealed elevated protein levels in all tested patients, with mild pleocytosis in 62.5%. At nadir, the modified Rankin Scale (mRS) ranged from 3 to 5. Immunomodulatory therapy was administered in 8 of 9 patients (88.9%), leading to notable functional improvement (mRS 1–2) in five cases. ICI rechallenge was attempted in two patients, neither of whom experienced recurrence. No deaths were directly attributed to irPRN.

**Conclusion:**

IrPRN is a rare but distinct complication of ICI therapy, typically presenting with a demyelinating neurophysiological pattern and favorable response to immunotherapy. Early recognition and treatment are critical to improving functional outcomes. Rechallenge may be feasible in selected cases without recurrence.

**Abbreviations**
AIDP: Acute inflammatory demyelinating polyneuropathy
ASCO: American Society of Clinical Oncology
CMAPs: Compound muscle action potentials
CNS: Central nervous system
CSF: Cerebrospinal fluid
CTLA‐4: Cytotoxic T‐lymphocyte‐associated protein 4
EMG: Electromyography
GBS: Guillain–Barré syndrome
ICIs: Immune checkpoint inhibitors
irAEs: Immune‐related adverse events
irPRN: Immune‐related polyradiculoneuropathy
IVIg: Intravenous immunoglobulin
mRS: Modified Rankin Scale
NCS: Nerve conduction studies
nirAEs: Immune‐mediated neurological adverse events
PD‐1: Programmed cell death protein 1
PNS: Peripheral nervous system
SEPs: Somatosensory evoked potentials
SNAPs: Sensory nerve action potentials

## 1 Introduction

Immune‐mediated neurological adverse events (nirAEs) secondary to immune checkpoint inhibitors (ICIs) are rare but potentially severe complications that can affect both the central nervous system (CNS) and the peripheral nervous system (PNS) (Farina et al. 2023; Marini et al. 2021; Zhao et al. 2021). Understanding their pathophysiology, clinical presentation, and therapeutic management is essential for optimizing the safety and efficacy of ICI‐based therapies.

The incidence of nirAEs is estimated to range between 1% and 3% (Farina et al. 2023). Within this spectrum, neurological syndromes affecting the PNS are more common than those involving the CNS, accounting for approximately 59% to 75% of cases (Farina et al. 2023; Marini et al. 2021). Following myositis and myasthenia gravis, the most frequent PNS‐nirAE is the immune‐related polyradiculoneuropathy (irPRN), which closely resembles Guillain–Barré‐like syndrome (Marini et al. 2021).

Although numerous irPRN/Guillain–Barré‐like syndrome case reports have published, larger detailed cohort studies are still lacking. Both Chen et al. (2019) and Dubey et al. (2019) presented series compiling various immune checkpoint inhibitor‐associated neuropathies, with acute polyradiculoneuropathies being among the most frequently reported subtypes—18 out of 23 cases in the former and 6 out of 19 in the latter. However, a comprehensive evaluation of their clinical and neurophysiological characteristics, therapeutic strategies, and clinical outcomes is still needed to enhance understanding and optimize management of these conditions.

This study aims to analyze the clinical and electrodiagnostic presentation, therapeutic management, and clinical outcomes of a cohort of patients with irPRN secondary to ICI therapy.

## 2 Methods

We conducted a single‐center retrospective descriptive observational study of all patients aged 18 and older who were diagnosed with irPRN secondary to ICI therapy at our institution between May 2018 and July 2024.

irPRN was defined as an acute or subacute onset polyradiculoneuropathy confirmed by neurophysiological findings secondary to ICI therapy, clinically resembling a Guillain–Barré‐like syndrome.

In accordance with the Consensus Disease Definition (Guidon et al. 2021), we considered nirAEs as neurological symptoms that began within 12 months of the last ICI infusion, after exclusion of other potential etiologies through a tailored diagnostic work‐up.

Our group recently published a study centered exclusively on the electrophysiological features PNS‐irAEs (Lainez et al. 2025). While some patients from that study are also included in the present work, the current study specifically focuses on a detailed clinical characterization of the subgroup diagnosed with immune‐related polyradiculoneuropathy (irPRN). Other types of PNS‐irAEs—such as myositis, myasthenia gravis, multineuritis, and sensory neuronopathies—were excluded from this analysis.

### 2.1 Clinical Data

A comprehensive review of medical records was conducted to collect demographic and clinical data, including cancer type, duration of ICI therapy, other treatments received, and clinical outcomes.

Their clinical outcomes were assessed based on the modified Rankin Scale (mRS) at three time points: before the onset of nirAE, at its most severe stage (nadir), and post‐management (6 months).

### 2.2 Electrodiagnostic Studies

Examinations included nerve conduction studies (NCS), specifically orthodromic sensory nerve action potentials (SNAPs) recorded proximal to the wrist crease for the median nerve, with stimulation of the middle finger using a ring electrode, and for the ulnar nerve with ring electrode stimulation of the fifth finger. The antidromic superficial radial sensory nerve response was also recorded over the wrist at the anatomical snuffbox. Antidromic measurements of the superficial peroneal and sural SNAPs were performed by recording at the ankle and stimulating the nerves 100–140 mm proximal to the recording site. Motor studies were therefore conducted to assess compound muscle action potentials (CMAPs) and F‐waves in the median, ulnar, tibial, and peroneal nerves. NCS were performed using standard techniques (Preston and Shapiro 2021), with recordings obtained from the abductor pollicis brevis, abductor digiti minimi, extensor digitorum brevis, and abductor hallucis muscles. Tibial H‐reflex studies were performed on the soleus muscles using as well standard techniques. All patients underwent NCS in at least three limbs.

Additionally, needle electromyography (EMG) was conducted in two to three limbs, with a minimum of two muscles assessed in the lower limbs.

Somatosensory evoked potentials (SEPs) were recorded following stimulation of the right tibial nerve using a bipolar surface electrode at the ankle. Two or three sets of 200–500 responses were averaged using monopolar subcutaneous needle electrodes for scalp derivations (Cz‐Fz). Cortical responses (P37/N45) were also recorded.

Electrodiagnostic evaluations were performed using Viking devices (Natus Neurology Inc./Nicolet Biomedical Inc., USA). Skin temperature was maintained above 32°C for all studies.

The types of ICI‐related neuropathy were classified as primary demyelinating, primary axonal, and equivocal according the published criteria for acute demyelinating neuropathy (Hadden et al. 1998).

#### 2.2.1 Standard Protocol Approvals, Registration, and Patient Consents

The study received approval from the bioethics committee (EOM(AG)040/2024(6298)).

## 3 Results

### 3.1 Demographic and Clinical Characteristics

The study cohort comprised nine patients diagnosed with immune‐mediated irPRN secondary to ICI therapy. The demographic, and ICIs characteristics are summarized in Table 1. The mean patient age was 67.9 years (range: 59–79 years), with a predominance of males (66.7%). The most commonly treated malignancy was non‐small cell lung cancer, representing three out of nine cases.

**TABLE 1:** Demographic, tumor, and ICIs characteristics of the sample.

	Patient 1	Patient 2	Patient 3	Patient 4	Patient 5	Patient 6	Patient 7	Patient 8	Patient 9
Age (years)	59	68	59	77	62	62	74	79	71
Sex (M/F)	F	M	F	F	F	M	M	M	M
Tumor type	Non‐small cell lung cancer	Clear cell renal cell carcinoma	Pleural mesothelioma	Non‐small cell lung cancer	Breast	Gastric adenocarcinoma	Hepatocellular carcinoma	Melanoma	Non‐small cell lung cancer
Previous chemotherapies	No	No	Cisplatin + pemetrexed	Cisplatin + pemetrexed	Docetaxel + trastuzumab	1st‐L cisplatin + 5‐fluorouracil 2nd‐L Docetaxel 3rd‐L Irinotecan	No	No	No
ICI	Pembrolizumab	Pembrolizumab	Nivolumab + ipilimumab	Pembrolizumab	Pembrolizumab	Sasanlimab	Pembrolizumab + quavonlimab	Nivolumab + ipilimumab	Pembrolizumab
ICI‐class	PD‐1	PD‐1	PD‐1 + CTLA‐4	PD‐1	PD‐1	PD‐1	PD‐1 + CTLA‐4	PD‐1 + CTLA‐4	PD‐1
Latency from ICI initiation (weeks)	2	6	14	24	4	20	6	11	3
ICI cycle	1	3	2	4	1	7	2	4	2
Other irAEs (Y/N)	No	Yes	No	Yes	No	No	No	Yes	Yes
Description other irAEs (irAE—PNP interval days)*	—	Encephalitis (—27 days) Hypophysitis (—68 days)	—	Pneumonitis (+45 days)	—	Encephalitis (+14 days)	—	Hepatitis (—5 days) Hypophysitis (—14 days)	Myasthenia gravis—myositis syndrome (simultaneous onset)

ICI: immune checkpoint inhibitors; M/F: male / female; PD‐1: programmed cell death 1, PD‐L1: programmed cell death ligand; CTLA‐4, cytotoxic T‐lymphocyte antigen 4, Y/N: yes/no.

*Time interval between other irAEs and irPRN onset (days; positive values indicate occurrence before PNP onset, negative values after PNP onset.

Four patients had received prior chemotherapy, whereas the remaining five initiated ICI therapy without previous cytotoxic treatment. All patients were treated with PD‐1 inhibitors, either as monotherapy (66.7%) or in combination with CTLA‐4 inhibitors (33.3%). There were no patients treated with CTLA‐4 monotherapy. The median latency from ICI initiation to the onset of irPRN symptoms was 6 weeks (range: 2–24 weeks). The majority of patients developed irPRN within the first 4 cycles of ICI therapy (median: 2 cycles, range: 1–7 cycles).

### 3.2 Concurrent Immune‐Related Adverse Events (irAEs)

Concurrent irAEs were documented in four cases, with two patients experiencing multiple irAEs simultaneously. These included encephalitis (2 patients), hypophysitis (2 patients), pneumonitis (1 patient), hepatitis (1 patient), hypothyroidism (1 patient), and myasthenia gravis–myositis syndrome (1 patient).

### 3.3 Clinical Presentation of irPRN

The clinical characteristics of the sample are summarized in Table 2. The clinical presentation of irPRN was characterized by symmetric limb muscle weakness in all patients (100%, 9/9). Lower limb weakness was predominant, affecting seven patients (77.8%), while two patients (22.2%) exhibited weakness in all four limbs. Sensory disturbances, including numbness, paresthesia, or sensory ataxia, were reported in eight patients (88.9%). All cases exhibited reduced or absent deep tendon reflexes.

**TABLE 2:** Clinical and CSF characteristics of the sample.

	Patient 1	Patient 2	Patient 3	Patient 4	Patient 5	Patient 6	Patient 7	Patient 8	Patient 9
**Symptoms**									
Limb muscle weakness	Yes (symmetric, 4 limbs)	Yes (symmetric, lower limbs)	Yes (symmetric, lower limbs)	Yes (symmetric, lower limbs)	Yes (symmetric, 4 limbs)	Yes (symmetric, lower limbs)	Yes (symmetric, lower limbs)	Yes (symmetric, lower limbs)	Yes (symmetric, 4 limbs)
Numbness/paresthesia/sensory ataxia	Yes	No	Yes	Yes	Yes	Yes	Yes	Yes	Yes
Ocular weakness	No	No	No	No	No	No	No	No	Yes*
Bulbar weakness	No	No	No	No	No	No	No	No	Yes*
Respiratory involvement	No	No	No	No	No	No	No	No	Yes*
Reflex in affected limbs	Absent	Absent	Present (2/5)	Absent	Absent	Absent	Absent	Absent	Absent
Latency from onset to nadir (days)	28	28	14	14	>90	7	14	10	21
**CSF finding**					Not performed				
CSF – Protein (mg/dL)	110	200	46	81	—	61	95	160	594
Cell (cel/µL)	0	72	10	2	—	78	13	28	0
Glucose (mg/dL)	63.7	105	54	57	—	65	54	51	87

CSF: cerebrospinal fluid.

*Ocular weakness, bulbar dysfunction, and respiratory involvement aligned with associated myasthenia gravis–myositis syndrome phenotype.

Ocular weakness, bulbar dysfunction, and respiratory involvement were observed exclusively in Patient 9, whose clinical presentation aligned with the myasthenia gravis–myositis syndrome phenotype. However, this patient also exhibited severe sensorimotor impairment in the limbs, suggesting an overlapping irPRN. No clinical signs suggestive of cranial neuropathy were observed in any other patient.

The median latency from symptom onset to nadir was 14 days (range: 7–90 days). In all cases except for Patient 5, disease progression reached its nadir within 8 weeks, indicating a relatively rapid clinical decline in most patients.

### 3.4 Cerebrospinal Fluid (CSF) Findings

CSF analysis was performed in eight patients (88.9%). Elevated CSF protein levels were observed in all tested patients, with a median concentration of 110 mg/dL (range: 46–594 mg/dL). Mild pleocytosis (<50 cel/µL) was detected in 5 of the eight cases (62.5%), while the remaining three showed normal cell counts (<5 cel/µL). CSF glucose levels were within normal limits in all cases (median: 57 mg/dL, range: 51–105 mg/dL).

### 3.5 Neurophysiological Findings

We analyzed electrodiagnostic studies from nine patients with neuropathy following ICI treatment (Table 3). The latency between symptom onset and the neurophysiological assessment ranged from 1 to 18 weeks (mean: 5.9 weeks). In eight of these cases, a demyelinating polyradiculoneuropathy was the predominant finding. Table 4 summarizes the neurophysiological findings.

**TABLE 3:** Electrodiagnostic results.

			**CMAPs**	**SNAPs**				
Demyelinating neuropathies		**Time between symptoms onset and Edx test**	**Number of demyelinating nerves**	**Number of nerves with decreased amplitude**	**H Reflex**	**SEP: P39 Lat (ms)**	**Needle EMG**	**Follow‐up Edx studies**
	Patient 1	3 weeks	4/4: Med, Uln, R Per and L Tib	2/4: Med and Uln (Sural sparring)	NR	NR	Mild‐moderate axonal loss without SA in 4 limbs	1 m: reduction in sural, peroneal, and tibial nerves amplitude. Normalization of F‐wave responses in the LL
	Patient 2	4 weeks	5/6: Med, Uln, L Per and R&L Tib	2/5: Med and Uln (Sural sparring)	A	A	Mild‐moderate axonal loss, distal predominance (D>I) with SA in the R TA	13 m: reduction in the R Per and L&L Tib amplitude, EMG: abundant SA in the distal musculature of all 4 limbs, with improved SEP lat
	Patient 3	4 weeks	3/5: Med and L&R Tib	1/5: Superficial peroneal	Increased Lat	Increased 52.6	Mild diffuse myogenic changes without SA	1 m: favorable evolution. 15 months: normalization of electroneurographic parameters, although increased SEP lat persists
	Patient 5	12 weeks	4/5: Med, Uln, L&R Tib (CPE A)	5/5: Med, Uln, Rad and L&L Sural	A	A	Severe bilateral distal denervation with SA in L TA and R gastr.	10 m: improvement in the amplitude of Uln and Sural SNAPs and Med and Uln CMAPs, and in Per and Tib DML, reappearance of SEP response
Axonal neuropathies			**Number of nerves with decreased amplitude**	**Number of nerves with decreased amplitude**				
	Patient 9	3 weeks	3/5: R Per and L&R TIb	2/4: L&R sural	NR	NR	Mild proximal myogenic involvement and severe axonal loss with diffuse SA	3.5 m: improvement in the amplitude of Med and Uln CMAPs, with resolution of myogenic signs
Equivocal			**Number of demyelinating nerves**	**Number of nerves with decreased amplitude**				
	Patient 4	2 weeks	0/4	1/4: Uln	NR	NR	Moderate‐severe diffuse axonal loss in the LL (L>R) with SA in the L TA	Not follow‐up
	Patient 6	6 weeks	1/5: R Per	0/5	Increased Lat	Increased 55	Minimal diffuse myogenic involvement without SA	1 m: improvement in the amplitude of the sural nerves, the CV of Med, Uln, Per and Tib CMAPs, and the lat of SEPs
	Patient 7	18 weeks	1/4: L Tib	2/4: L&R Sural	Increased Lat	Increased 50.6	Moderate‐severe distal axonal loss with SA	5 m: no significant changes in electroneurographic parameters, EMG, or SEP
	Patient 8	1 week	1/4: L Tib	0/4	NR	Increased 52.4	Mild proximal myogenic involvement and mild‐moderate distal axonal loss without SA	32 m: slight decrease in SNAP amplitude (except Uln) and CMAPs of Per and Tib. Normalization of F and H responses, and EMG. No changes in SEP lat

Edx: electrodiagnostic; CMAPs: compound motor action potentials, SNAPs: sensory nerve action potentials; SEP: somatosensory evoked potentials; Lat: latency; EMG: electromyography, Med: median nerve; Uln: ulnar nerve; Per; peroneal nerve; Tib: tibial nerve; L: left; R: right; NR: not recorded; A: absent; SA: spontaneous activity; TA: tibial anterior muscle; Gastr: gastrocnemius muscle; LL: lower limbs; DML: distal motor latency; CV: conduction velocity; m: month.

**TABLE 4:** Management and outcomes of the sample.

	Patient 1	Patient 2	Patient 3	Patient 4	Patient 5	Patient 6	Patient 7	Patient 8	Patient 9
mRS (before nirAE, nadir, post‐management)	1 → 5 → 1	1 → 5 → 3	1 → 3 → 1	1 → 4 → 2	1 → 5 → 5	1 → 5 → 2	1 → 3 → 1	1 → 4→ 1	1 → 5 → 4
**Management**									
Immunomodulatory/ suppressive treatment	IVIg (2 g/kg) – 1 cycle + Oral prednisone (1 mg/kg) tapering	1 g methylprednisolone 5 days + plasmapheresis (5 sessions)	IVIg (2 g/kg) – 1 cycle + Oral prednisone (1 mg/kg) tapering	IVIg (2 g/kg) – 1 cycle + Oral prednisone (1 mg/kg) tapering	No treatment ((neurological evaluation performed 4 months after symptom onset)	Oral prednisone (1 mg/kg) tapering	Oral prednisone (1 mg/kg) tapering	IVIg (2 g/kg) – 1 cycle + 1 g methylprednisolone 5 days + Oral prednisone (1 mg/kg) tapering	IVIg (2 g/kg) – 3 cycles + 1 g methylprednisolone 5 days + plasmapheresis (5 sessions) + Oral prednisone (1 mg/kg) tapering
ICI withdrawal	Yes	Yes	Yes	Yes	Yes	Yes	Yes	Yes	Yes
ICI rechallenge	No	No	Yes	No	No	No	Yes	No	No
nirAE outcome	Relapse 15 month later after starting new line chemotherapy (inhibitor RET kinase)	Initial improvement, but relapse at 4 weeks. IVIg (2 g/kg) – 1 cycle was administered + oral prednisone (1 mg/kg) tapering. No more relapses	After 4 months, immunotherapy was restarted. Four cycles were administered with no recurrence, but with progression of the oncologic disease	No relapses	No relapses	No relapses	After 5 months, immunotherapy was restarted. New cycles were administered with no recurrence, but with progression of the oncologic disease after 5 months	No relapses	No relapses
Follow‐up (months)	36 months (death secondary to PD)	11 months (alive)	34 months (alive)	6 months (death secondary to PD)	18 months (death secondary to PD)	11 months (death secondary to PD)	14 months (death secondary to PD)	48 months (alive)	6 months (alive)

mRS: modified Rankin Scale; post‐management: approximately at 6 months; IVIg: intravenous Immunoglobulin; ICI: immune checkpoint inhibitors; PD: progression of disease.

According to Hadden's criteria (Hadden et al. 1998), four patients had primary demyelinating neuropathy (involving two or more nerves with demyelination), one had axonal neuropathy, and the remaining four had equivocal forms (fewer than two nerves with demyelination).

In the demyelinating group, abnormal motor conduction and demyelination were widely distributed across various segments of the peripheral nerves tested, revealing significant heterogeneity among individuals. The most common abnormalities included prolonged distal motor latencies, slow conduction, and prolonged or absent F‐wave latencies. Regarding SNAPs, 3 out of 4 patients showed diminished amplitudes in at least two nerves. Two of them exhibited a sural sparing pattern (normal sural SNAP amplitude with abnormal ulnar SNAP amplitude) (Uncini et al. 2017). In 3 of the 4 patients in whom H‐reflex responses and SEPs were performed, abnormal responses were observed.

In the equivocal group, three of four patients had one motor nerve showing electrophysiological evidence of demyelination, along with abnormal H‐reflexes and increased latency of SEPs. The remaining patient showed only moderate‐to‐severe active denervation in the lower limbs on needle EMG, indicating radicular involvement.

The only patient in the primary axonal group showed decreased amplitudes in both motor and sensory nerves, particularly in the lower limbs. There were no prior predisposing factors for developing polyneuropathy, and the patient had not received prior chemotherapy.

Follow‐up electrodiagnostic studies were performed on eight patients at intervals ranging from 1 to 32 months after the initial evaluation. Four patients, including the one with a primary axonal phenotype, showed improvement in electroneurographic parameters; one of them achieved complete normalization. However, in the case of axonal neuropathy, axonal loss in the lower limbs did not improve. Two patients exhibited stable findings, while two showed worsening neuropathy, characterized by a reduction in the amplitude of compound muscle action potentials (CMAPs) and sensory nerve action potentials (SNAPs), despite improvements in F‐wave responses or somatosensory evoked potential (SEP) latencies.

Additionally, in four cases, needle EMG revealed myogenic patterns associated with polyneuropathy. Except for Case 9, which was associated with a myasthenia gravis–myositis syndrome, the remaining three cases had creatine kinase levels within the normal range. All patients with coexistent myopathy showed resolution of myogenic changes in follow‐up electrodiagnostic studies.

### 3.6 Therapeutic Strategies

Management and clinical outcomes of the sample are summarized in Table 2.

Management primarily involved intravenous immunoglobulin (IVIg), corticosteroids, and plasmapheresis. One patient did not receive immunomodulatory treatment due to delayed evaluation and clinical considerations.

All treated patients received corticosteroid therapy: 5 out of 8 were given oral prednisone (1 mg/kg), and 3 out of 8 received 1 g of methylprednisolone for 5 days. In 7 of the 8 patients treated with corticosteroids, an oral corticosteroid tapering regimen was subsequently implemented. The patient who did not undergo tapering was the only one who experienced an early relapse.

Concomitantly with corticosteroids, 4 patients received IVIg treatment (2 g/kg). Three of them received a single cycle, while the patient with an associated myasthenia gravis–myositis syndrome underwent a total of three IVIg cycles. Plasmapheresis was used in only two cases: Patient 2 (in combination with corticosteroids) and Patient 9 (as part of triple therapy with corticosteroids and IVIg).

ICI therapy was discontinued in all cases. Two patients received ICI rechallenge following irPRN resolution. Both tolerated the treatment without polyradiculoneuropathy recurrence; however, they subsequently experienced progression of their underlying malignancy.

The management strategies and outcomes of the sample are summarized in Table 1.

### 3.7 Clinical Outcomes

Prior to the onset of nirAE, all patients had a modified Rankin Scale (mRS) score of 1, indicating minimal functional impairment. At the nadir of their condition, scores worsened significantly, ranging from 3 to 5, consistent with moderate to severe disability.

At follow‐up, approximately 6 months after symptom onset, most patients demonstrated substantial recovery, although the degree of improvement varied. Six patients achieved complete or near‐complete functional recovery, with mRS scores of 1 or 2. In contrast, three patients continued to experience moderate disability, with scores between 3 and 5. The patient who did not receive immunomodulatory treatment (Patient 5) had the poorest prognosis, with a persistent mRS score of 5 at the 6‐month follow‐up.

Importantly, no patients died as a direct result of irPRN.

### 3.8 Long‐Term Follow‐Up

Follow‐up duration ranged from 6 to 48 months. At the last evaluation, four patients remained alive, though two experienced disease progression. The remaining five patients died, all due to progression of their primary malignancy rather than complications related to irPRN.

Relapses were infrequent but occurred in two cases. Patient 1 experienced a late relapse in the context of starting a new chemotherapy regimen with a RET kinase inhibitor. Patient 2 experienced an early relapse and was notably the only case in which an oral prednisone taper was not administered after initial high‐dose corticosteroid therapy. This patient was subsequently treated with IVIg (2 g/kg) and oral prednisone (1 mg/kg), with no further relapses observed during follow‐up.

## 4 Discussion

**FIGURE 1:**
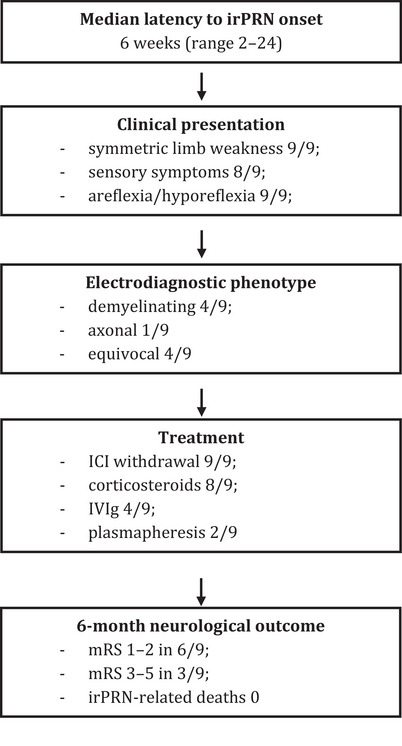
Visual summary of clinical course, treatment, and outcomes in irPRN.

This study provides a comprehensive characterization of irPRN secondary to ICI therapy, focusing on its clinical presentation, neurophysiological findings, therapeutic approaches, and clinical outcomes. While irPRN is a rare complication, its potential severity and impact on functional status underscore the importance of early recognition and timely intervention. To enhance clarity, we have added a figure (Figure 1) summarizing the clinical course, treatments, and outcomes of the included cases.

The median latency from ICI initiation to irPRN onset (6 weeks) is slightly shorter than previously reported timelines, including a prior systematic review focused on irPRN (8 weeks) (Li et al. 2021) and the reported latency for other PNS‐irAEs (8.8–10 weeks) (Bruna et al. 2020; Rossi et al. 2023).

Regarding the ICI class, a previous study suggested that irPRN cases were associated with a higher proportion of patients treated with anti‐CTLA4 (alone or combined with anti‐PD(L)1), accounting for 60.1% of cases (Farina et al. 2023). In contrast, our study observed a higher proportion of patients treated exclusively with anti‐PD(L)1, representing 66.7% of cases.

Although prior chemotherapy exposure has been suggested as a potential risk factor for ICI‐induced nirAEs (Yan et al. 2023), more than half of our patients (55.6%) had no prior treatment. This finding suggests that irPRN can occur independently of previous oncologic therapies, reinforcing the hypothesis of a direct ICI‐mediated immune mechanism. Moreover, the predominance of demyelinating features in the electrophysiological findings primarily reflects immune‐mediated neurotoxicity rather than residual effects from previous treatments, despite the absence of baseline studies prior to ICI initiation. Notably, the only patient presenting with an axonal polyneuropathy had not undergone chemotherapy either.

Clinically, irPRN predominantly manifested as symmetric limb weakness, primarily affecting the lower extremities, along with sensory disturbances and absent or diminished reflexes in nearly all patients. While these features align with the classical presentation of post‐infectious GBS, irPRN differs in its more pronounced and exclusive involvement of lower limb weakness.

Concomitant irAEs were observed in 44.4% of patients, at a frequency closely resembling that reported in the literature (Farina et al. 2023). Notably, four patients developed other nirAEs, including encephalitis, hypophysitis, or MG/myositis, which may suggest an immune response targeting specific nervous system antigens. The concomitant occurrence of MG/myositis and irPRN had been previously described in a prior case (Chen et al. 2017).

At the neurophysiological level, demyelination was confirmed in the majority of patients with irPRN. Although the electrodiagnostic Hadden's criteria suggestive of AIDP were met in only four patients, three of the four additional patients classified as equivocal exhibited electrophysiological evidence of demyelination in one motor nerve. If other electrodiagnostic parameter, such as an abnormal H‐reflex and/or prolonged SEPs latency, indicative of radicular involvement (Van Den Bergh et al. 2010) had been considered, all of these patients would likely have been classified as having demyelinating neuropathy. These results are consistent with the hypothesis that compact myelin is likely the primary target of ICI‐related neuropathy, although not as evident as in classic AIDP (Albarrán et al. 2022; Chen et al. 2019). In our cohort, the radicular region (F‐waves, H‐reflex, and SEP) was the most sensitive in the electrodiagnostic tests for RPN diagnostic. We did not find conduction block or abnormal temporal dispersion in any patient.

One patient presented with sensory and motor axonal neuropathy, with no evidence of demyelination on electrophysiological studies. These findings suggest that, in some cases, ICIs may also induce axonal degeneration, aligning with previous clinical observations (Bruna et al. 2020; Chen et al. 2019; Marini et al. 2021).

CSF analysis revealed elevated protein levels in all tested patients, consistent with inflammatory neuropathies and further supporting an immune‐mediated pathogenesis. Mild pleocytosis was observed in 62.5% of cases, distinguishing these cases from typical GBS, where pleocytosis has been reported in approximately 30% of cases (Al‐Hakem et al. 2023). This suggests potential differences in the underlying immunopathogenic mechanisms between irPRN and classical post‐infectious GBS.

The rapid and severe functional deterioration observed in the majority of patients highlights the critical importance of prompt therapeutic intervention, including ICI discontinuation and initiation of immunomodulatory treatment. Notably, within our cohort, the patient with the poorest 6‐month prognosis was the case in which irPRN was detected late, ICI therapy was not discontinued, and immunomodulatory treatment was not administered in a timely manner. Although corticosteroids are generally not recommended for GBS, the European and American oncology societies' clinical practice guidelines recommend their use in ICI‐induced neuropathies (Haanen et al. 2022; Schneider et al. 2021). Our findings suggest that combination therapy may be beneficial in irPRN cases; however, further studies are needed to optimize treatment protocols and identify the most effective therapeutic strategies. Additionally, our data support the use of an oral corticosteroid taper, as the only patient who experienced an early relapse was the one who received a 5‐day methylprednisolone mega dose without an oral taper.

ICI discontinuation was uniformly implemented across our cohort, aligning with current management strategies (Haanen et al. 2022; Schneider et al. 2021). According to the ASCO guidelines, the decision to resume ICI therapy should take into account the previous tumor response, the severity and type of the initial irAE, the time to resolution of the irAE, and the availability of alternative therapies. Recent evidence suggests that some patients may tolerate ICI rechallenge after resolution of the neurological event (Eldani et al. 2024; Farina et al. 2023). In our study, two patients underwent ICI reintroduction without recurrence of irPRN, though their oncologic disease progressed, limiting further assessment. These cases highlight the delicate balance between managing immune‐related toxicity and maintaining oncologic efficacy, emphasizing the need for individualized risk‐benefit assessments in patients requiring ongoing immunotherapy.

In our cohort, no cases of death associated with nirAEs were observed, which contrasts with findings from other reviews that have documented mortality linked to this neurological complication (Farina et al. 2023; Li et al. 2021). However, we believe this discrepancy may be attributed to the early diagnosis of 8 out of the 9 cases, with their prognosis resembling that of GBS. Furthermore, we believe that PNS‐nirAEs should be assessed by a team of expert neurologists and neurophysiologists, capable of accurately distinguishing irPRN from myositis/MG, conditions that are associated with a more unfavorable prognosis and require distinct therapeutic management (Rossi et al. 2023).

This study has several limitations. First, its retrospective design and small sample size limit the generalizability of our findings. Larger, multicenter studies are needed to further elucidate risk factors, optimize treatment strategies, and assess long‐term outcomes. Additionally, while many patients demonstrated meaningful neurological recovery, the broader impact of irPRN on quality of life and oncologic treatment continuation remains unclear and warrants further investigation.

## 5 Conclusion

This study highlights the clinical, electrodiagnostic, and therapeutic characteristics of irPRN secondary to ICI therapy, demonstrating its rarity and potential severity. Early diagnosis and prompt immunomodulatory treatment appears to be critical for optimizing functional outcomes, as timely interventions lead to significant recovery in most patients. The study also underscores the heterogeneity in clinical presentations and neurophysiological patterns, with a predominance of demyelinating features. While corticosteroids and intravenous immunoglobulin are effective treatments, further research is needed to optimize therapeutic strategies and identify the most beneficial protocols. Given the complexity of these cases, expert neurologists should play a key role in distinguishing irPRN from other immune‐related neuropathies to ensure appropriate management and improve patient outcomes.

Future research should focus on refining diagnostic criteria, assessing the safety of ICI rechallenge, and exploring novel therapeutic strategies to mitigate the long‐term morbidity associated with irPRN.

## Author Contributions

**Lainez E**.: conceptualization, investigation, writing – original draft, writing – review and editing, visualization, validation, methodology, software, formal analysis, project administration, resources, supervision, data curation. **Gratacòs‐Viñola M**.: conceptualization, investigation, writing – original draft, methodology, validation, data curation, supervision. **Llauradó A**.: conceptualization, investigation, writing – original draft, methodology, validation, visualization, writing – review and editing, funding acquisition, software, formal analysis, project administration, resources, supervision, data curation. **Raguer N**.: funding acquisition, conceptualization, validation, methodology, formal analysis, data curation, resources, supervision. **Juntas‐Morales R**.: supervision, resources, data curation, formal analysis, validation, methodology, funding acquisition, conceptualization. **Sanchez‐Tejerina D**.: investigation, methodology, data curation.

## Funding

The authors have nothing to report.

## Conflicts of Interest

The authors declare no conflicts of interest.

## Ethics Statement

The study received approval from the bioethics committee (EOM(AG)040/2024(6298)). We confirm that we have read the Journal's position on issues involved in ethical publication and affirm that this report is consistent with those guidelines.

## Data Availability

The data that support the findings of this study are available on request from the corresponding author. The data are not publicly available due to privacy or ethical restrictions.
